# Mild clinical features of isolated methylmalonic acidemia associated with a novel variant in the *MMAA* gene in two Chinese siblings

**DOI:** 10.1186/s12881-018-0635-4

**Published:** 2018-07-11

**Authors:** Yiming Lin, Chunmei Lin, Weihua Lin, Zhenzhu Zheng, Mingya Han, Qingliu Fu

**Affiliations:** 1 Neonatal Disease Screening Center of Quanzhou, Quanzhou Maternal and Children’s Hospital, 700 Fengze Street, Quanzhou, 362000 Fujian Province China; 2 Genuine Diagnostics Company Limited, 859 Shixiang West Road, Hangzhou, 310007 Zhejiang Province China

**Keywords:** Isolated methylmalonic aciduria, Novel variant, *MMAA* gene, Next-generation sequencing

## Abstract

**Background:**

Methylmalonic acidemia (MMA) is an autosomal recessive inherited disorder caused by complete or partial deficiency of the enzyme methylmalonyl-CoA mutase (mut0 enzymatic subtype or mut– enzymatic subtype, respectively); a defect in the transport or synthesis of its cofactor, adenosyl-cobalamin (cblA, cblB, or cblD-MMA); or deficiency of the enzyme methylmalonyl-CoA epimerase. The cblA type of MMA is very rare in China. This study aimed to describe the biochemical, clinical, and genetic characteristics of two siblings in a Chinese family, suspected of having the cblA-type of MMA.

**Methods:**

The Chinese family of Han ethnicity of two siblings with the cblA-type of MMA, was enrolled. Target-exome sequencing was performed for a panel of MMA-related genes to detect causative mutations. The influence of an identified missense variant on the protein’s structure and function was analysed using SIFT, PolyPhen-2, PROVEAN, and MutationTaster software. Moreover, homology modelling of the human wild-type and mutant proteins was performed using SWISSMODEL to evaluate the variant.

**Results:**

The proband was identified via newborn screening (NBS); whereas, her elder brother, who had not undergone expanded NBS, was diagnosed later through genetic family screening. The younger sibling exhibited abnormal biochemical manifestations, and the clinical performance was relatively good after treatment, while the older brother had a mild biochemical and clinical phenotype, mainly featuring poor academic performance. A novel, homozygous missense c.365T>C variant in exon 2 of their *MMAA* genes was identified using next-generation sequencing and validated by Sanger sequencing. Several different types of bioinformatics software predicted that the novel variant c.365T>C (p.L122P) was deleterious. Furthermore, three-dimensional crystal structure analysis revealed that replacement of Leu122 with Pro122 led to the loss of two intramolecular hydrogen bonds between the residue at position 122 and Leu188 and Ala119, resulting in instability of the MMAA protein structure.

**Conclusions:**

The two siblings suspected of having the cblA-type of MMA showed mild phenotypes during follow-up, and a novel, homozygous missense variant in their *MMAA* genes was identified. We believe that the clinical features of the two siblings were associated with the *MMAA* c.365T>C variant; however, further functional studies are warranted to confirm the variant’s pathogenicity.

**Electronic supplementary material:**

The online version of this article (10.1186/s12881-018-0635-4) contains supplementary material, which is available to authorized users.

## Background

Methylmalonic acidemia (MMA) is an autosomal recessive inherited disorder that is characterized by the abnormal accumulation of methylmalonyl-CoA and methylmalonic acid in body fluids, which is caused by either a defect in methylmalonyl-CoA mutase (MCM, EC 5.4.99.2) or a defect in the transport or synthesis of its cofactor, adenosyl-cobalamin (AdoCbcl) [1, 2]. The clinical presentation of MMA varies, but it is characterized by recurrent vomiting, lethargy, seizures, metabolic acidosis, brain damage, and developmental delay. According to the characteristic biochemical findings, MMA can be divided into isolated MMA without hyperhomocysteinemia and combined MMA with hyperhomocysteinemia. Additionally, complementation studies have revealed the presence of at least eight different MMA subtypes, including mut0/mut- (complete or partial deficiency, respectively), cblA-D, cblF, cblJ, and cblX [3, 4]. In China, 60–80% of patients with MMA have combined MMA, i.e., cblC (MIM 277400), classic cblD (MIM 277410), cblF (MIM 277380), and cblJ (MIM 614857); in particular, the cblC type is more common [5]. Isolated MMA includes four subtypes: mut0/mut- (MIM 251000), cblA (MIM 251100), cblB (MIM 251110), and cblD variant 2 (MIM 277410) [6, 7]. Most of the reported Chinese patients with isolated MMA are of the mut0/mut- type, and the less prevalent cblA-type of MMA has not been well documented. In the present study, we described the biochemical, clinical, and genetic characteristics of two siblings in a Chinese family, suspected of having the cblA-type of MMA.

## Methods

### Subjects and auxiliary analysis

**Fig. 1:**
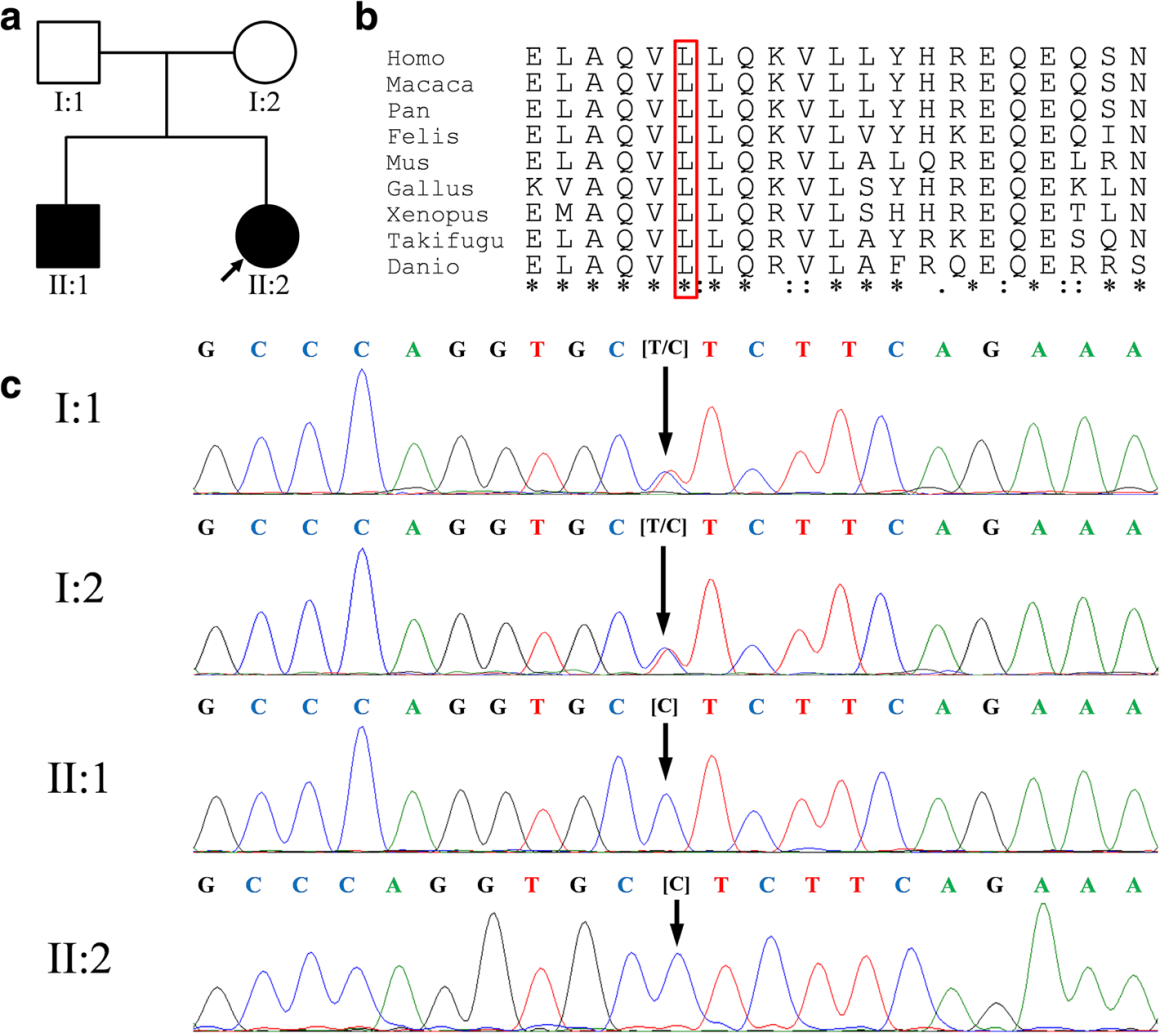
**a** Pedigree of isolated methylmalonic acidemia with *MMAA* variants. The arrow denotes the proband, and affected family members are indicated by filled black symbols. **b** Multiple sequence alignment by use of ClustalX. The presence of a leucine residue at position 122 (highlighted with a box) was highly conserved across different species. **c** Validation of the *MMAA* gene mutation by Sanger sequencing. The proband and her affected brother are homozygous for c.365T>C, and their parents are heterozygotes (the variant is indicated by a black arrow)

This study investigated a two-generation Chinese family, containing four members of Han ethnicity, from the Fujian Province (Fig. 1a). The proband (II:2) was identified via newborn screening (NBS), while, her older brother (II:1), a 10-year-old boy, was not diagnosed then because the NBS program at that time only included tests for phenylketonuria (PKU) and congenital hypothyroidism (CH); he was diagnosed later because of a positive family history. The parents were non-consanguineous healthy individuals, and there was no family history of MMA or other genetic metabolic disorders. A hundred healthy newborns with normal, expanded neonatal disease screening results from our centre were enrolled as control subjects. The concentrations of blood propionylcarnitine (C3) and the C3 to acetylcarnitine (C2) ratio (C3/C2) in the dried blood spots of both patients were analysed using tandem mass spectrometry (MS/MS) (Waters, ACQUITY TQD, Milford, MA, USA). Their urine samples were then collected for organic acid analysis using gas chromatography-mass spectrometry (GC–MS) (Agilent, 7890B/5977A, Santa Clara, CA, USA). Meanwhile, the levels of serum total homocysteine, folic acid, and vitamin B12 in plasma were also analysed. In addition, physical and mental evaluations, including the development quotient (DQ) test and brain magnetic resonance imaging (MRI) analysis, were conducted. The study was approved by the ethics committee of The Maternal and Children’s Hospital of Quanzhou. Written informed consent was obtained from the parents of all of the patients and control subjects, who agreed to join this study, with the intent of using the medical data for scientific research and publication.

### DNA isolation and next-generation sequencing (NGS)

Genomic DNA was extracted from the dried blood spots obtained from the proband and her family members, as well as the control subjects, using Qiagen Blood DNA mini kits (Qiagen, Hilden, Germany), following the manufacturer’s protocol. DNA was quantified using a Qubit® dsDNA HS Assay Kit (Invitrogen, Carlsbad, CA, USA). DNA samples of the proband were used for NGS. The target sequences of a panel of MMA-related genes (*MUT*, *MMAA*, *MMAB*, *MMACHC*, *MMADHC*, *LMBRD1*, *ABCD4*, and *HCFC1*) were enriched using multiplex polymerase chain reaction (PCR). The library concentration and amplicon size were determined using an Agilent High Sensitivity DNA Kit (Agilent, Santa Clara, CA, USA). The libraries were sequenced with an Illumina Miseq sequencer (Illumina, San Diego, CA, USA) for paired-end reads of 150 bp. After Miseq sequencing, high-quality reads were retrieved from the raw reads by filtering out the low quality reads and adaptor sequences, using the Trimmomatic program (http://www.usadellab.org/cms/index.php?page¼trimmomatic). Single nucleotide polymorphisms (SNPs) and insertions or deletions (InDels) were identified using the SAMtools program (http://www.htslib.org/).

### Variant filtering and bioinformatics analysis

The identified variants were checked for their presence in disease databases, such as the Human Gene Mutation Database (http://www.hgmd.cf.ac.uk/ac/index.php), ClinVar (https://www.ncbi.nlm.nih.gov/clinvar/), the Leiden Open Variation Database (http://www.lovd.nl/3.0/home), dbSNP (https://www.ncbi.nlm.nih.gov/projects/SNP/), the 1000 Genome Project (http://www.1000genomes.org/) database, and the ExAC consortium (http://exac.broadinstitue.org/), to confirm their novelty. The identified novel missense mutations were further assessed for possible pathogenicity using PolyPhen-2 (http://genetics.bwh.harvard.edu/pph2/), SIFT (http://sift.jcvi.org/), PROVEAN (http://provean.jcvi.org/index.php), and MutationTaster (http://www.mutationtaster.org/). Multiple amino acid sequences were then extracted from the National Center for Biotechnology Information (NCBI) and aligned using ClustalX (http://www.clustal.org/clustal2) to evaluate the evolutionary conservation of the variants. Additionally, homology modelling was used to build three-dimensional (3D) models of MMAA, which was accomplished using the Swiss Model Workspace [8] with the PDB entry code 2www. The PDB files were submitted to Swiss-pdb Viewer 4.0 to view the 3D-structure [9].

### Sanger sequencing

The newly identified c.365T>C variant was validated by Sanger sequencing of samples from all of the family members. *MMAA* exon 2 and its flanking sequences were amplified by PCR using the following primers: forward, 5′-TAGAATATGGGGGAAACA-3′ and reverse, 5′-CACACAAAAAAGACTGACC-3′. Sanger sequencing was performed using an ABI Prism 3500 Genetic Analyzer (Applied Biosystems, Foster City, CA, USA), and the results were analysed using DNASTAR software (http://www.dnastar.com/).

## Results

### Biochemical and clinical analysis

In the family with members suspected of having MMA, the proband had significantly elevated levels of blood C3 and urinary methylmalonic and methylcitric acids, and an increased ratio of C3/C2, with plasma homocysteine in the normal range. The concentrations of C3 and the C3/C2 ratios showed an upward trend at pretreatment. The patient was therefore treated with intramuscular hydroxocobalamin (1 mg/day, 2–3 times/week) and L-carnitine (50–100 mg/kg/day, oral administration) on day 50, which caused a favourable metabolic response, such that the blood C3 and urine MMA concentrations decreased remarkably. However, the patient did not consistently use the medication and the levels of the metabolic markers increased again. The results of the cerebral MRI were normal, and the most recent DQ test was correlated to the mid-low level. The patient showed normal physical growth at the latest follow up in April 2018.

The proband’s older brother exhibited a slightly elevated C3 concentration and C3/C2 ratio during the first acidemic attack, with elevated methylmalonic excretion, but undetectable methylcitric acid in his urine. However, in the absence of treatment, the C3 concentration returned to the normal range, and the C3/C2 ratio approached the upper limit after two months. The main characteristic of the patient was poor academic performance. The boy, who was in the fifth grade of primary school, could only read a few words, implying some degree of mental retardation. In addition, the parents complained that he rarely ate meat, fish, or other high-protein foods. The patient showed normal physical development during follow-up, and his brain MRI also showed no structural abnormalities. Detailed information on the biochemical, clinical, and molecular characteristics of the siblings are summarized in Table 1.

**Table 1:** Biochemical, clinical, and genetic characteristics of the siblings

Patient no.	Gender	Current age	Age at test	MS/MS analysis			Methylmalonic acid (mmol/mol creatinine)^d^	Methycitrate (mmol/mol creatinine)^e^	Homocysteine (μmol/L)^f^	DQ	Cerebral MRI	Genotype
				C3 (μmol/L)^a^	C3/C2^b^	C3/C0^c^						
II:1	Male	10 y, 8 m	9 y, 9 m	4.96	0.53	0.26	32.51	0.24		ND	Normal	Homozygous for c.365T>C variant
			9 y, 11 m	1.22	0.31	0.12	13.29	0.76	6.07			
II:2	Female	1 y, 2 m	4 d (NBS)	7.05	0.71	0.85						Homozygous for c.365T>C variant
			12 d	8.14	1.3	0.81	56.46	5.49				
			45 d	9.93	1.54	1.01						
			50 d^g^	19.76	1.08	0.57						
			2 m	6.3	0.28	0.11	8.88	3.95	6.5		Normal	
			3 m	3.95	0.24	0.08	39.39	1.97				
			5 m	4.93	0.21	0.1	15.24	1.35				
			8 m	8.49	0.29	0.21	48.97	1.88		92.8		
			1y, 1 m	7.76	0.38	0.19	42.71	1.25		84.1		

*C3* Propionylcarnitine, *C2* Acetylcarnitine, *C0* Free carnitine, *NBS* Newborn screening, *y* Year, *m* Month, *d* Day, *DQ* Development quotient, *ND* Not determined, *MRI* Magnetic resonance imaging

^a^normal range: 0.50–4.50 μmol/L

^b^normal range: 0.01–0.20

^c^normal range: 0.02–0.20

^d^normal range: 0.3–3.6 mmol/mol creatinine

^e^normal range: 0.2–1.1 mmol/mol creatinine

^f^normal range: 0-15 μmol/L

^g^Treatment was commenced on day 50

### Mutation analysis

**Fig. 2:**
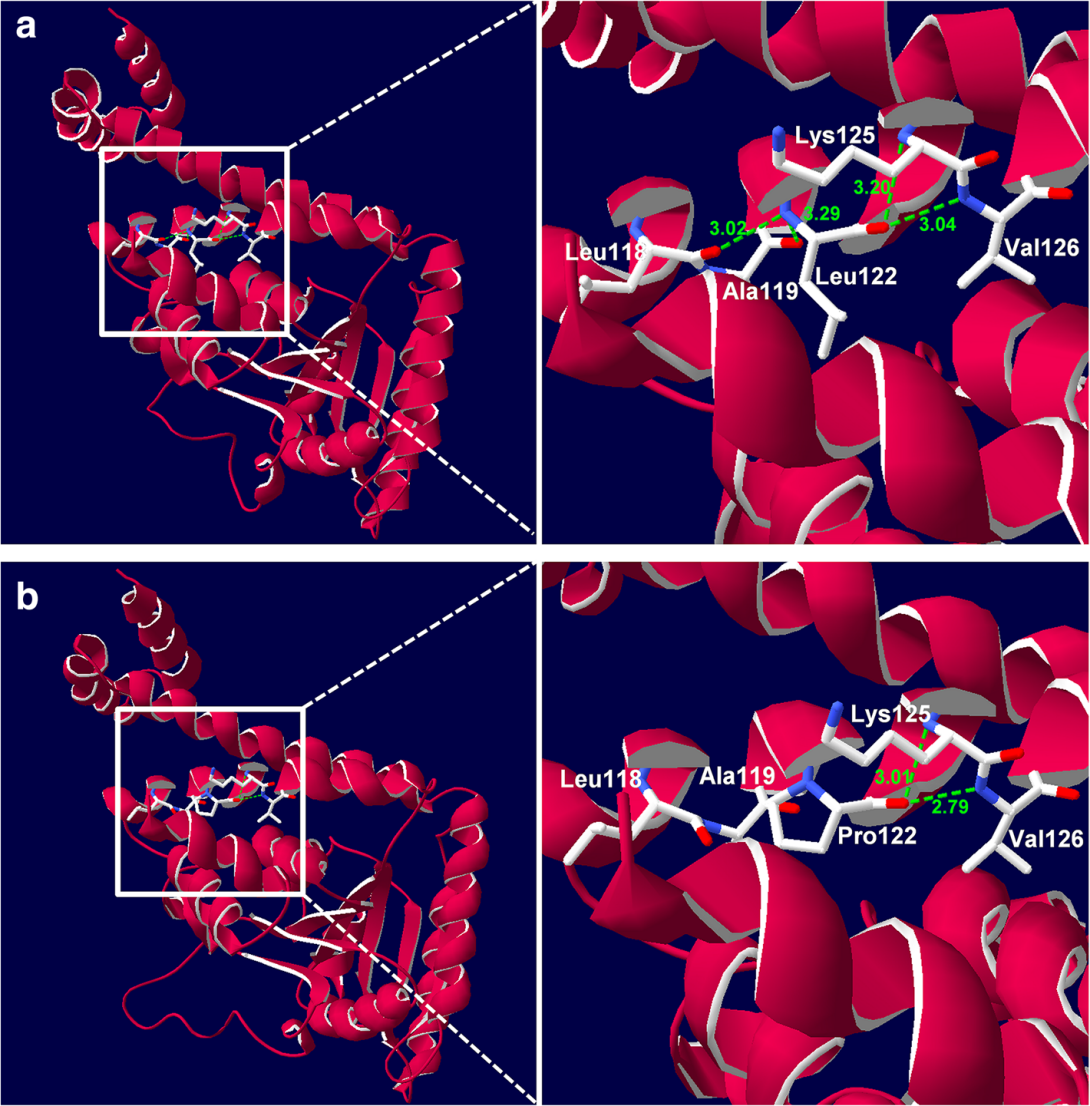
Structural analysis of the wild-type and MMAA mutant products using three-dimensional modelling. Green dashed lines represent hydrogen bonds, and the green Arabic numbers show the hydrogen bond distances. **a** A segment of the MMAA structure showing Leu122 hydrogen bonding with Leu188, Ala119, Lys125, and Val126. **b** A segment showing that Pro122 lost two intramolecular hydrogen bonds, one with Leu188 and one with Ala119, leading to instability of the protein structure

A summary of the target-enriched next generation sequencing data is presented in Additional file 1: Table S1. The mean read depth was 254.03×, and 100% of the targeted bases were covered at depths of ≥20×. Following further variant filtering analysis, we identified a novel, homozygous missense variant, c.365T>C, in exon 2 of the *MMAA* gene, as the potential disease-causing variant. This novel variant has not been reported in any previous study; the allele frequency in the ExAC database was 8.253e-06 in all populations, and it was not listed in HGMD, ClinVar, LOVD, dbSNP, or the 1000 Genome project database (Additional file 2: Table S2). Sanger sequencing validated that the proband and her older brother were homozygous for this variant and that their parents were heterozygous carriers (Fig. 1c). This variant was absent in the control population. Several different types of bioinformatics software predicted that the novel variant was a deleterious mutation (Table 2). The leucine at position 122 was found to be a highly conserved amino acid residue across several different species (Fig. 1b). Additionally, Swiss-pdb Viewer 4.0 revealed that the replacement of Leu with Pro at position 122 led to the loss of two intramolecular hydrogen bonds, one with Leu188 and one with Ala119, likely resulting in instability of the MMAA protein structure (Fig. 2). Taken together, the c.365T>C variant was considered deleterious and likely to be pathogenic.

**Table 2:** In silico prediction of the *MMAA* c.365T>C variant

Software	Score	Prediction effect
PolyPhen-2	0.999	Probably damaging
SIFT	0	Affecting protein Function
PROVEAN	−5.879	Deleterious
MutationTaster	1	Disease causing

## Discussion

MMA is a common, organic acidemia in China. Its prevalence shows regional differences and is estimated to vary from 1:48000 to 1:250000. The incidence of MMA in the mainland of China remains unclear [10, 11]. The cblC type is responsible for a large number of MMA cases [12]; whereas, the cblA-type of MMA is very rare, and only two Chinese patients with the cblA-type of MMA have been diagnosed to date [13, 14].

This study reported two siblings suspected to have the cblA-type of MMA in a Chinese family. The proband was identified using NBS, and the abnormal NBS and subsequent urine organic acid results were suggestive of MMA. For further identification, target-exome enriched next generation sequencing was performed for a panel of MMA-related genes, and a novel, homozygous c.365T>C variant in the *MMAA* gene was identified. The proband was treated with continuous intramuscular hydroxocobalamin and L-carnitine, because of the obvious abnormal biochemical manifestations. The proband had normal physical and mental development during the short period of follow-up; therefore, we classified this patient as having vitamin B_12_-responsive MMA. Genetic family screening revealed that the proband’s older brother was also homozygous for the same variant. The brother had not undergone expanded NBS ten years ago and had not been diagnosed previously. He showed normal development and mainly featured poor academic performance, which indicated some degree of mental retardation that may be at least partially due to suffering from MMA. It is possible that the patient’s mild phenotype is related to his long-term lack of high-protein foods. However, because there were no remarkable abnormalities, the parents refused the relevant treatment for their children, but did agree to follow-up under our recommendations. The initial MS/MS analysis showed a small increase in the blood C3 and the C3/C2 ratio. C3 returned to the normal range when tested again two months later; a slightly increased C3/C2 ratio, near the cut-off limitation, was still observed, which might be easily misdiagnosed, and suggested the higher specificity of the C3/C2 ratio. In general, the patients’ clinical phenotypes have been relatively mild to date, with good outcomes, which is consistent with previous reports that patients with the cblA-type of MMA have relatively mild phenotypes, among the isolated types of MMA patients [15–17].

The 17.1 kb *MMAA* gene is located on chromosome 4q31.1–q31.2 and contains seven exons that encode a protein of 418 amino acids [18]. To date, more than 60 mutations in the *MMAA* gene have been reported to cause isolated MMA, and some are specific to certain ethnicities [19–21]. For example, c.433C>T is common in the European population and is always accompanied by the presence of the SNP c.820-110A>G [22], while c.503delC is prevalent in Japanese patients [23]. In China, Liu et al. [13] reported the first case of the cblA-type of MMA in a patient with acute encephalopathy, induced by vaccination, who was compound heterozygous for c.650T>A and c.742C>T in the *MMAA* gene. In the same year, Sun et al. [14] reported another patient with the cblA-type of MMA, who was compound heterozygous for the *MMAA* mutations c.586C>T and c.898C>T. In this study, we identified a novel homozygous *MMAA* c.365T>C variant that led to the conversion of a leucine at position 122 to proline. This variant has not been reported in PubMed or related databases. A variety of bioinformatics software predictions and amino acid conservation analysis showed that the variant had a deleterious effect on the gene or gene product. In addition, 3D-modelling analysis showed that the substitution of leucine with proline led to the loss of two intramolecular hydrogen bonds, one with Leu188 and one with Ala119, resulting in instability of the MMAA protein structure. According to the guidelines published by the American College of Medical Genetics Association of Clinical Genetics (ACMG) [24], although the c.365T>C variant did not demonstrate sufficient evidence to be classified as pathogenic and was instead classified as a variant of unknown significance (VUS), we believe that the identified novel variant was associated with the pathogenesis of MMA in the siblings. The unequivocal pathogenicity of this variant requires more evidence, such as further functional studies and more case reports. Therefore, the current study has the following limitations: (i) as the patients do not have the typical clinical consequences of the cblA disorder and the c.365T>C variant lacks functional studies, the diagnosis may not be clear; and (ii) due to the fact that the targeted NGS gene panel did not cover deep intron regions, it remains possible that the true causative variants were not identified.

## Conclusions

In this study, we described the biochemical, clinical, and genetic characteristics of two siblings of a Chinese family, suspected of having the cblA-type of MMA. The siblings showed mild phenotypes during follow-up, and a novel, homozygous missense variant in the *MMAA* gene was identified. However, further functional studies are warranted to evaluate the pathogenicity of the c.365T>C variant to clarify the diagnosis.

## Additional files

Additional file 1: **Table S1.** Summary of targeted gene sequencing data in the proband. (DOC 29 kb)

Additional file 2: **Table S2.** Shortlist of seven variants identified in MMA-related genes by targeted NGS. (DOC 35 kb)

## Abbreviations

3D: Three-dimensional
ACMG: American College of Medical Genetics Association of Clinical Genetics
AdoCbcl: Adenosyl-cobalamin
DQ: Development quotient
GC-MS: Gas chromatography-mass spectrometry
InDels: Insertions or deletions
MCM: Methylmalonyl-CoA mutase
MMA: Methylmalonic acidemia
MRI: Magnetic resonance imaging
MS/MS: Tandem mass spectrometry
NBS: Newborn screening
NGS: Next-generation sequencing
PCR: Polymerase chain reaction
SNPs: Single nucleotide polymorphisms
VUS: Variant of unknown significance
