# Supplementary material for: Mild clinical features of isolated methylmalonic acidemia associated with a novel variant in the MMAA gene in two Chinese siblings
Source: BMC Med Genet. 2018 Jul 11;19:114. doi: 10.1186/s12881-018-0635-4 (PMC6042273; doi:10.1186/s12881-018-0635-4)
Supplement: Supplementary file 1 — Table S1. Summary of targeted gene sequencing data in the proband. (DOC 29 kb) [file 12881_2018_635_MOESM1_ESM.doc]

**Additional file 1: Table S1.** Summary of targeted gene sequencing data in the proband.

| Raw reads | 81283 |
| --- | --- |
| Unique mapping reads ratio | 83.91% |
| Mean read depth on target | 254.03 |
| Fraction of target covered with at least 1× | 100% |
| Fraction of target covered with at least 20× | 100% |
| Fraction of target covered with at least 50× | 99.06% |
| Fraction of target covered with at least 100× | 93.4% |
