# Supplementary material for: Mild clinical features of isolated methylmalonic acidemia associated with a novel variant in the MMAA gene in two Chinese siblings
Source: BMC Med Genet. 2018 Jul 11;19:114. doi: 10.1186/s12881-018-0635-4 (PMC6042273; doi:10.1186/s12881-018-0635-4)
Supplement: Supplementary file 2 — Table S2. Shortlist of seven variants identified in MMA-related genes by targeted NGS. (DOC 35 kb) [file 12881_2018_635_MOESM2_ESM.doc]

**Additional file 2: Table S2.** Shortlist of seven variants identified in MMA-related genes by targeted NGS.

| No. | Mutation name | #Chrom | Position | Refer | Alternative | Gene | RefSeq | Region | Genotype | CytoBand | Freq in  ExAC | Freq in  1000Genome | SNP138 |
| --- | --- | --- | --- | --- | --- | --- | --- | --- | --- | --- | --- | --- | --- |
| 1 | c.321G>A | chr1 | 45973928 | G | A | *MMACHC* | NM_015506 | exonic | het | 1p34.1 | 0.4664 | 0.47524 | rs2275276 |
| 2 | c.453G>A | chr2 | 150432976 | C | T | *MMADHC* | NM_015702 | exonic | hom | 2q23.2 | 0.8681 | 0.759585 | rs11545261 |
| **3** | **c.365T>C** | **chr4** | **146560656** | **T** | **C** | ***MMAA*** | **NM_172250** | **exonic** | **hom** | **4q31.21** | **8.253e-06** | **NA** | **ND** |
| 4 | c.716T>A | chr12 | 109994870 | A | T | *MMAB* | NM_052845 | exonic | het | 12q24.11 | 0.5095 | 0.513379 | rs9593 |
| 5 | c.1407T>A | chr6 | 70407465 | A | T | *LMBRD1* | NM_018368 | exonic | het | 6q13 | 0.392 | 0.384784 | rs12648 |
| 6 | c.910G>A | chr14 | 74759477 | C | T | *ABCD4* | NM_005050 | exonic | het | 14q24.3 | 0.3285 | 0.270367 | rs4148077 |
| 7 | c.3490T>C | chrX | 153220360 | A | G | *HCFC1* | NM_005334 | exonic | het | Xq28 | 0.5008 | 0.58596 | rs1051152 |

NA: not available

ND: no data

hom: homozygous variant

het: heterozygous variant
